# EMG biofeedback combined with rehabilitation training may be the best physical therapy for improving upper limb motor function and relieving pain in patients with the post-stroke shoulder-hand syndrome: A Bayesian network meta-analysis

**DOI:** 10.3389/fneur.2022.1056156

**Published:** 2023-01-10

**Authors:** Sisi Feng, Mingzhi Tang, Gan Huang, JuMei Wang, Sijin He, Duo Liu, LiHua Gu

**Affiliations:** ^1^Yunnan University of Traditional Chinese Medicine, Kunming, China; ^2^Department of Rehabilitation, Kunming Hospital of Traditional Chinese Medicine, The Third Affiliated Hospital of Yunnan University of Chinese Medicine, Kunming, China

**Keywords:** stroke, shoulder-hand syndrome (SHS), physical therapy, rehabilitation training, network meta analyses

## Abstract

**Background:**

Post-stroke shoulder-hand syndrome (SHS), although not a life-threatening condition, may be the most distressing and disabling problem for stroke survivors. Thus, it is essential to identify effective treatment strategies. Physical therapy is used as a first-line option for treating SHS; however, it is unclear which treatment option is preferred, which creates confusion in guiding clinical practice. Our study aims to guide clinical treatment by identifying the most effective physical therapy interventions for improving clinical symptoms in patients with post-stroke SHS using Bayesian network meta-analysis.

**Methods:**

We conducted a systematic and comprehensive search of data from randomized controlled trials using physical therapy in patients with SHS from database inception to 1 July 2022. Fugl-Meyer Upper Extremity Motor Function Scale (FMA-UE) and pain visual analog score (VAS) were used as primary and secondary outcome indicators. R (version 4.1.3) and STATA (version 16.0) software were used to analyze the data.

**Results:**

A total of 45 RCTs with 3,379 subjects were included, and the intervention efficacy of 7 physical factor therapies (PFT) combined with rehabilitation training (RT) was explored. Compared with the control group, all the PFT + RT included were of statistical benefit in improving limb motor function and pain relief. Also, our study indicated that EMG biofeedback combined with RT (BFT + RT) [the surface under the cumulative ranking curve (SUCRA) = 96.8%] might be the best choice for patients with post-stroke SHS.

**Conclusion:**

EMG biofeedback combined with rehabilitation training may be the best physical therapy for improving upper limb motor function and relieving pain in patients with post-stroke SHS according to our Bayesian network meta-analysis results. However, the above conclusions need further analysis and validation by more high-quality RCTs.

**Systematic review registration:**

www.crd.york.ac.uk/prospero/, identifier: CRD42022348743.

## Introduction

Shoulder-hand syndrome (SHS), also known as reflex sympathetic dystrophy (RSD), is mainly characterized by local pain, limitation of upper extremity movement with swelling, abnormal skin temperature, and skin changes. As a common complication in stroke patients with hemiplegia, usually occurring in patients within 1–3 months after stroke, SHS is a crucial factor affecting the recovery of motor function in the upper extremity of patients (1, 2). Nonetheless, failure to provide timely and unreasonable interventions may prolong SHS patients' recovery, even resulting in permanent deformities of the shoulder, upper limb, and finger, which may seriously affect their daily lives and prognoses (3).

Modern medicine has not yet elucidated the pathogenesis of SHS after stroke. It may be related to reflex sympathetic nerve damage that leads to a series of inflammatory and autoimmune reactions, and the generation of abnormal cytokines (4). Furthermore, limb paralysis impairs the circulation of body fluids in the upper limb of patients with stroke, leading to stasis edema in the affected limb and shoulder-hand pump dysfunction. This may be an essential reason for the pathogenesis of SHS (5). Various microtraumas, such as repeated blood draws, intravenous injections, or inappropriate active and passive motion, might also contribute to or exacerbate SHS (6, 7).

The current clinical treatment of post-stroke SHS focuses on reducing pain while maintaining and restoring function for patients. Drug treatment mainly includes oral anti-inflammatory, analgesic, immune modulating (such as glucocorticoids and non-steroidal anti-inflammatory drugs) and anticonvulsant and antidepressant drugs or injection of stellate nerve block, steroid hormone joint cavity injection closure, intravenous bisphosphonate injection, intradermal injection of botulinum toxin, and other invasive drugs (8–11). While pharmacological treatment is convenient and quick, its long-term use will produce side effects such as infection, poor compliance, and drug resistance. Consequently, it can only relieve some clinical symptoms but cannot fundamentally control and treat the occurrence and development of SHS (12). The treatment guidelines (13) highlight that since pain and limb dysfunction are the main clinical problems associated with SHS, early physical therapy intervention is the basis and first-line choice for SHS treatment. In addition, most experts, even those who use more invasive interventional techniques, agree that effective treatment should emphasize functionally focused interventions, particularly physical therapy that aims at normalizing the function of the affected limb and alleviating problems associated with disuse (14).

Physical therapy, as the main body of rehabilitation treatment, includes exercise therapy based on rehabilitation training and physical factor therapy (PFT) with various physical factors (sound, light, cold, heat, point, magnetic, and water) as the primary means. Although exercise therapy is an indispensable intervention to SHS treatment, some patients still refuse to use the affected limb because of severe pain or experience huge emotional stress. It makes it difficult for them to stick to the treatment and thus reduces its expected efficacy (15, 16). PFT (as a safe and effective alternative therapy) not only provides anti-inflammatory, analgesic, neuromuscular excitation, and spasticity relief *via* the mediating impact of electrotherapeutic stimulation but is also easily accepted by patients due to the comfort of the treatment procedure (17). Various PFT techniques are often combined with rehabilitation training (RT) in clinical practice to treat SHS, and its efficacy is good. However, the advantages of different PFT vary, and there are no relevant guidelines to rank their efficacy on patients with SHS, which confuses the clinical guiding practice. Therefore, we aim to conduct a comprehensive review of RCTs of different physical factor therapies combined with rehabilitation training for the treatment of post-stroke SHS using Bayesian network meta-analysis (NMA), expecting to find the optimal physiotherapy regimen to guide clinical practice.

## Materials and methods

This study was conducted in accordance with the Preferred Reporting Items for Systematic Reviews and Meta-Analyses (PRISMA) extended statement (18). This NMA has been registered on the International prospective register systematic reviews (PROSPERO) with the registration number CRD42022348743. No ethical approval or patient consent was required for this study since all analyses were conducted based on previously published studies.

### Search strategy

We conducted a comprehensive search of the following databases: Web of Science, PubMed, EMBASE, Cochrane Central Controlled Trials, China Knowledge Network (CNKI), Wanfang database, VIP database, and China Biomedical Literature Database (CBM). With no restrictions on language or publication time, we identified the randomized controlled trials (RCTs) on the observation of the efficacy of physiotherapy on post-stroke SHS published before 1 July 2022.

By combining medical subject headings (MeSH) with free words using Boolean logic operators, we integrated the following terms for a comprehensive search: “stroke,” “cerebral infarction,” “cerebral hemorrhage,” “shoulder-hand syndrome,” “reflex sympathetic dystrophy,” “complex localized pain syndrome type I,” “electrotherapy,” “low-frequency pulsed electrical stimulation,” “neuromuscular electrical stimulation,” “transcutaneous electrical nerve stimulation,” “ultrasound,” “ultrashort wave,” “infrared therapy,” “laser therapy,” “wax therapy,” “wet-hot compress,” “air wave pneumatic therapy,” “hyperbaric oxygen,” “magnetotherapy,” “transcranial magnetic stimulation,” “biofeedback therapy,” “electromyographic biofeedback therapy,” “rehabilitation training,” and “randomized controlled trial.” Moreover, we manually screened the reference lists in the relevant meta-analyses and reviews to minimize the omission of literature that meets the inclusion criteria. Taking the PubMed search as an example, details of the search strategy are shown in Supplementary Table 1. Two independent authors (SSF and MZT) processed the screening records using Endnote 20 literature management software (Thompson ISI Research Soft, Philadelphia, Pennsylvania, USA). Disagreements in this process were resolved by discussion or by a third author (LHG).

### Selection and exclusion criteria

The inclusion of studies meeting the criteria should be based on the PICOS framework:

**Population:** Patients were diagnosed with post-stroke SHS according to clear diagnostic criteria (19, 20), without restriction to gender or age.

**Intervention:** Acceptable treatment is mainly various physical factor therapy (PFT) combined with rehabilitation training (RT). PFT includes electrotherapy (ET), light therapy (LT), ultrasound therapy (UWT), conductive heat therapy (CHT), and pressure therapy (PT). As well as magnetotherapy (MT), which is based on transcranial magnetic stimulation, and biofeedback therapy (BFT), which is based on electromyography biofeedback (EMGBF) as the main intervention. However, there are no restrictions on the frequency, duration, and waveform of the above PFT.

Among them, ET contains low-frequency pulsed electrical stimulation, transcutaneous neuromuscular electrical stimulation, medium-frequency electrotherapy, and ultrashort wave; LT, infrared radiation and laser therapy; UWT, ultrasound and extracorporeal shock wave; CHT, Chinese herbal wet and hot compresses and wax therapy; PT, air pressure, air wave pressure therapy, and hyperbaric oxygen.

**Comparison:** RT alone or intercomparison between interventions.

**Outcomes:** Primary outcomes: Fugl-Meyer Upper Extremity Motor Function Scale (FMA-UE). Secondary outcomes: Visual analog score of pain (VAS).

**Study design:** Randomized controlled trials only. Non-randomized controlled studies, such as animal trials, reviews, systematic reviews, case-control studies, and study protocols, were excluded.

Based on the criteria set above, two authors (GH and JMW) independently screened the titles and abstracts to exclude duplicates and studies that did not meet the inclusion criteria. Subsequently, the eligible studies were reviewed in full. Any inconsistencies that arose during this period were decided by consensus.

### Data extraction and quality assessment

Following the Cochrane Consumer and Communications Review Group's data extraction template, we completed relevant data collection for eligible studies: including basic publication information (first author's name and year of publication), participant characteristics (total sample size, age, and duration of disease), interventions, duration of treatment, and quality of RCTs, among other relevant information.

The quality of each eligible study was assessed by two independent investigators (MZT and GH) using the Cochrane Risk of Bias Tool (21). A total of seven areas were covered (random sequence generation, allocation concealment, blinding of participants and personnel, blinding of outcome assessments, incomplete data on outcome data, selective reporting, and other biases). Each item was rated as unknown, low, or high risk of bias. The assessment was performed in Review Manager (version 5.4).

### Statistical analyses

According to the minimally informative prior distributions of the Bayesian random effects model (22), we first performed a conventional pair-wise meta-analysis by synthesizing the essential data from all the included studies. Evaluated effect sizes for each pair-wise treatment comparison in terms of continuous outcome, mean difference (MD) was calculated along with 95% credible intervals (CrIs) as the pooled relative effect and estimate uncertainly, respectively. As a visual representation of statistical heterogeneity, *I*^2^ statistic was tested to assess whether substantial heterogeneity existed. The values 25, 50, and 75% indicated mild, moderate, and high heterogeneity, respectively (18). To detect whether any bias was generated, a comparison-adjusted funnel plot was made as a concise description, and both were analyzed using the Egger test (23). We constructed a network plot for offering all the existing relationships, with distinct treatments expressed by different nodes and trials by lines joining appropriate nodes.

Network transitivity is the most crucial assumption underlying NMA, whose assessment would affect our further analysis directly (24). Therefore, to ensure the sufficient similarity of various treatment comparisons, which can provide valid indirect inferences, we evaluate the transitivity assumption by comparing the clinical and methodological characteristics, such as the characteristics of participants and experimental design, across all the included studies (25, 26). In order to simulate an accurate estimation of the statistical model, four parallel Markov chains were first established in the random selection state (27). Each chain generated 50,000 iterations. Due to the burn-in period, an initial 20,000 iterations were discarded to minimize the bias of initial values when the chain reached its target distribution (28). The Brooks-Gelman-Rubin diagnostic was used to evaluate the convergence of the models by visually inspecting the historical trajectory of trace combined with density plots (29) (see Supplementary Figure 1 for details). As the estimated probability of ranking the physical treatments, the surface under the cumulative ranking curve (SUCRA) was presented as a simple numerical summary statistic cumulative ranking probability plot for each treatment (30). SUCRA with a higher value denotes a greater likelihood of a given treatment being in the top rank or highly effective. In contrast, the value “zero” indicates that the treatment is sure to be the worst. Finally, to explore whether potential source inconsistency arises in our network, we use the “node splitting” technique, comparing direct and indirect evidence across the network (when *P* > 0.05 indicates that consistency arises) (31, 32). The above analyses were performed using the “Gemtc” package (version 1.0–1) and “rjags” (version 4–13) in R software (version 4.1.3), and STATA (version 16.0) software (StataCorp, College Station, TX, USA).

## Results

### Search process and baseline characteristics

We initially retrieved 735 literature studies, of which 343 were duplicates. After the screening of titles and abstracts, 286 documents were excluded. We reviewed the remaining 106 studies for full text; 6 studies were presented as case reports or study protocols; 2 studies were diagnosed with other types of disease; 11 studies did not adopt the method of random grouping; 19 studies did not meet the inclusion criteria for this study; 13 studies did not provide relevant outcome indicators for our analysis; 8 studies were not available in full text or had incomplete outcome indicators, and another 2 were duplicate published studies. Thus, 45 clinical randomized controlled trials that meet the inclusion criteria were finally included (33–78).

**Figure 1:**
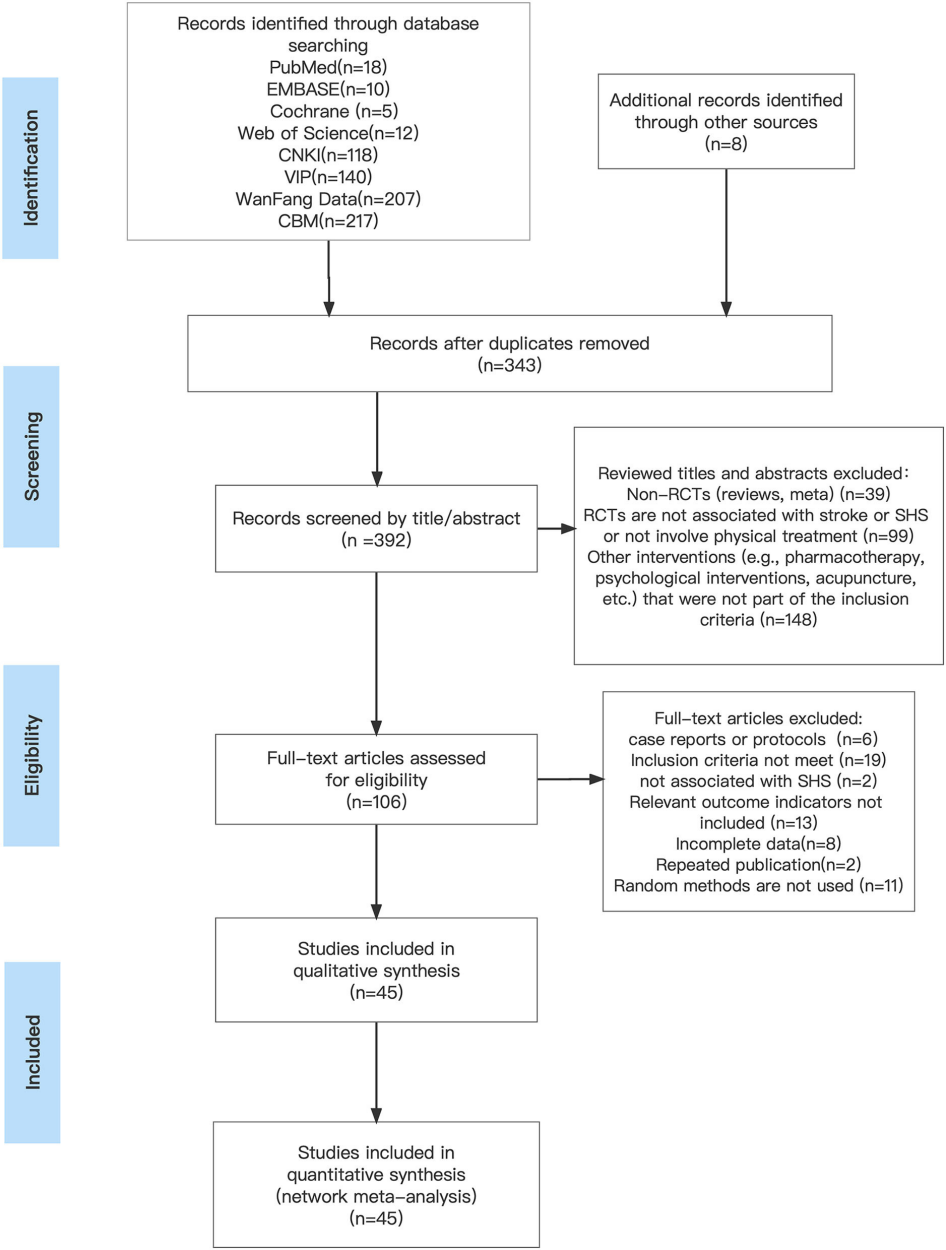
Literature screening process.

Figure 1 depicts the processing of the literature screening.

Table 1 summarizes key characteristics such as participant baseline information and interventions in detail. The included studies were from China, and the literature was published between 2008 and 2022. A total of 3,379 study participants were randomly assigned to either the trial or control group. Of these, 1,696 participants were included in the trial group of seven different physical factor therapies combined with rehabilitation training (BFT + RT, *n* = 135; CHT + RT, *n* = 352; PT + RT, *n* = 259; ET + RT, *n* = 379; MT + RT, *n* = 132; UWT + RT, *n* = 174; LT + RT, *n* = 265). The remaining 1,683 individuals were randomized into four control groups (CHT + RT, *n* = 69; ET + RT, *n* = 60; LT + RT, *n* = 81; RT, *n* = 1,473).

**Table 1:** Characteristics of included studies.

**Study ID**	**Participant**		**Age**	**Gender (M/F)**	**Interventions**		**Course**	**Outcome**
	**T**	**C**			**T**	**C**		
Zhang et al. (79)	100	100	T: 57.10 ± 1 0.88	T: 57/43	CHT + RT	RT	30d	FMA-UE
			C: 56.30 ± 10.72	C: 59/41				VAS
Zhao et al. (80)	51	50	T: 58.12 ± 2.41	T: 27/24	LT + RT	RT	28d	FMA-UE
			C: 57.89 ± 2.37	C: 27/23				
Tian et al. (81)	28	29	T: 65.90 ± 9.50	T: 15/13	MT + RT	RT	2w	FMA-UE
			C: 66.97 ± 10.51	C: 14/15				VAS
Wu (33)	28	28	T: 59.4 ± 10.7	T: 15/13	MT + RT	RT	27d	FMA-UE
			C: 58.5 ± 9.5	C: 15/13				VAS
Li et al. (34)	25	25	T: 62.28 ± 13.79	T: 22/3	UWT + RT	RT	4w	FMA-UE
			C: 61.68 ± 11.91	C: 20/5				VAS
Liu and Wang (35) (a)	50	50	T: 60.2 ± 10.8	T: 29/21	MT + RT	RT	2w	FMA-UE
			C: 62.7 ± 10.7	C: 27/23				VAS
Ren et al. (40)	40	40	T: 51.64 ± 7.47	T: 22/18	CHT + RT	RT	4w	FMA-UE
			C: 57.28 ± 10.66	C: 24/16				VAS
Chen and Zheng (43)	27	27	T/C: 63.8 ± 8.4	/	BFT + RT	CHT + RT	4w	FMA-UE
								VAS
Li and Lai (42)	40	40	T: 62.2 ± 8.9	T: 23/17	ET + RT	RT	4w	FMA-UE
			C: 61.7 ± 9.3	C: 24/16				VAS
Zhang et al. (41) (a)	30	30	T: 60.1 ± 7.31	T: 18/12	ET + RT	RT	4w	FMA-UE
			C: 59.1 ± 7.9	C: 19/11				VAS
Weng et al. (46)	30	30	T: 68.82 ± 3.34	T: 18/12	CHT + RT	RT	4w	FMA-UE
			C: 68.85 ± 3.36	C: 19/ 11				VAS
Li (50)	15	15	T: 48.7 ± 5.3	T: 10/5	PT + RT	RT	21d	VAS
			C: 46.5 ± 6.8	C: 8/7				
Wu et al. (51)	30	30	T: 54.5 ± 6.5	T: 18/12	PT + RT	RT	18d	FMA-UE
			C: 56.2 ± 7.6	C: 16/14				
Cai et al. (55)	37	33	T: 57.14 ± 3.99	T: 17/20	ET + RT	RT	47d	FMA-UE
			C: 58.36 ± 4.48	C: 16/17				VAS
Lin et al. (56)	42	42	T: 63.1 ± 8.3	T: 24/18	BFT + RT	CHT + RT	4w	FMA-UE
			C: 62.5 ± 9.2	C: 25/17				VAS
Li et al. (57) (a)	30	30	T: 64.7 ± 16.9	T: 19/11	UWT + RT	ET + RT	4w	FMA-UE
			C: 65.4 ± 17.3	C: 17/13				VAS
Hu et al. (65)	36	36	T: 61.2 ± 17.8	T: 20/16	PT + RT	RT	4w	FMA-UE
			C: 60.1 ± 18.2	C: 21/15				VAS
She et al. (64)	30	30	T: 57.39 ± 3.18	T: 18/12	ET + RT	RT	4w	FMA-UE
			C: 59.13 ± 4.53	C: 16/14				VAS
Zhao and Ma (66)	25	25	T: 63.3 ± 4.6	T: 18/7	LT + RT	RT	20d	FMA-UE
			C: 60.2 ± 5.8	C: 16/9				
Zhang et al. (67)	35	33	T: 61.4 ± 10.9	T: 20/15	ET + RT	RT	3w	VAS
			C: 60.8 ± 11.3	C: 20/13				
Liu and Dong (71)	20	20	T: 63.7 ± 11.4	T: 14/6	ET + RT	RT	3w	FMA-UE
			C: 62.8 ± 12.1	C: 13/7				VAS
Su and Chen (72)	30	30	T: 61	T: 19/11	PT + RT	RT	30d	FMA-UE
			C: 63	C: 18/12				VAS
Yang et al. (36)	31	31	T: 71.81 ± 9.95	/	ET + RT	RT	4w	VAS
			C: 72.42 ± 9.68					
Liu et al. (37) (b)	40	39	T: 63.38 ± 9.22	/	ET + RT	RT	2w	FMA-UE
			C: 64.21 ± 9.35					VAS
Qiao and Ding (39)	51	51	T: 53.45 ± 5.48	T: 22/29	CHT + RT	LT + RT	28d	FMA-UE
			C: 53.56 ± 5.34	C: 23/28				VAS
Gong et al. (45)	30	30	/	/	CHT + RT	LT + RT	21d	FMA-UE
								VAS
Guo and Ruan (62)	60	60	T: 63.1 ± 3.2	T: 36/24	CHT + RT	RT	3w	VAS
			C: 61.1 ± 2.6	C: 37/23				
Zhou et al. (49)	20	20	T: 63.71 ± 6.45	T: 16/4	ET + RT	RT	6w	FMA-UE
			C: 63.12 ± 6.89	C: 15/5				VAS
Yuan and Chen (59)	40	40	T: 51.73 ± 11.16	T: 24/16	PT + RT	RT	10d	FMA-UE
			C: 51.66 ± 11.01	C: 22/18				VAS
Shi et al. (61)	40	40	T: 52.73 ± 11.17	T: 24/16	ET + RT	RT	4w	FMA-UE
			C: 52.65 ± 10.03	C: 22/18				VAS
Guo and Ruan (62)	36	31	T: 52	T: 25/12	BFT + RT	RT	4w	FMA-UE
			C: 48	C: 19/12				VAS
Wang et al. (63)	40	40	T: 65.8 ± 12.6	T: 27/13	LT + RT	RT	4w	FMA-UE
			C: 66.3 ± 12.6	C: 23/17				VAS
Liu et al. (68)	46	46	T: 62.4 ± 9.6	T: 29/17	PT + RT	RT	4w	FMA-UE
			C: 61.4 ± 10.2	C: 28/19				VAS
Zhang and Huang (70)	45	45	T: 52.63 ± 9.67	T: 25/20	PT + RT	RT	15d	FMA-UE
			C: 51.26 ± 10.13	C: 26/19				VAS
Yang et al. (50)	56	56	T: 56.85 ± 10.7	T: 31/25	ET + RT	RT	14d	FMA-UE
			C: 56.72 ± 10.12	C: 29/27				
Tan (48)	41	41	T: 56.56 ± 3.34	T: 23/18	CHT + RT	RT	10d	FMA-UE
			C: 56.23 ± 3.16	C: 24/17				VAS
Bao et al. (38)	30	30	T: 63.32 ± 6.13	T: 16/14	UWT + RT	RT	4w	FMA-UE
			C: 64.82 ± 8.27	C: 16/14				VAS
Zhang et al. (44) (b)	29	29	T: 53.91 ± 5.33	T: 13/16	UWT + RT	RT	4w	FMA-UE
			C: 53.70 ± 5.73	C: 15/14				VAS
Zhang and Huang (47)	26	26	T: 51.31 ± 7.32	T: 16/10	MT + RT	RT	4w	FMA-UE
			C: 53.18 ± 9.40	C: 17/9				VAS
Liu et al. (74)	32	31	T: 58.84 ± 6.12	T: 17/15	PT + RT	RT	30d	FMA-UE
			C: 60.04 ± 5.95	C: 18/13				VAS
Wang et al. (60)	54	54	T/C: 55.27 ± 13.5	/	LT + RT	RT	4w	FMA-UE
Xue et al. (54)	30	30	T: 62.7 ± 5.4	T: 15/15	BFT + RT	RT	6w	FMA-UE
			C: 63.4 ± 6.7	C: 14/16				
Yan et al. (82)	30	30	T: 53.52 ± 15.32	T: 16/14	UWT + RT	RT	4w	FMA-UE
			C: 53.85 ± 15.13	C: 17/13				
Lu et al. (58)	80	80	T: 62.2 ± 4.9	T: 46/34	LT + RT	RT	4w	FMA-UE
			C: 63.4 ± 4.9	C: 44/36				VAS
Li et al. (57) (b)	30	30	T: 64.7 ± 16.9	T: 19/11	UWT + RT	ET + RT	2w	FMA-UE
			C: 65.4 ± 17.3	C: 17/13				VAS

ET, electrotherapy; LT, light therapy; UWT, ultrasonic wave therapy; CHT, conduction heat therapy; PT, pressure therapy; MT, magnetic therapy; BFT, biofeedback therapy; RT, rehabilitation training; FMA-UE, fugl-meyer upper extremity motor function scale; VAS, visual analog score of pain; C, control group; T, treatment group; d, day; w, week.

### Quality of included studies

Summary tables of individual and overall level quality assessments are detailed in Supplementary Figures 2, 3. All 45 studies (33–78) reported group randomization, but allocation concealment was unclear. Due to intervention limitations, only two studies (33, 40) adopted the single-blind method for participants; four studies (40, 59, 71, 75) evaluated the study results using the blind method. Five studies (36, 41, 45, 63, 65) reported detailed cause shedding. All 45 included studies reported on the pre-specified outcomes completely. In addition, two studies (44, 60) mentioned no adverse effects.

### Network analysis results

#### Primary outcome: FMA-UE

The preliminary conventional meta-analysis observed a high degree of heterogeneity in the FMA-UE score among studies (*I*^2^ = 88.2%, *P* = 0.000). The adjusted funnel plots showed a relatively symmetrical distribution of studies on both sides of the inverted funnel. However, some smaller studies are distributed below and outside the inverted funnel, suggesting the possible presence of publication bias (Supplementary Figure 1). An additional Egger's test was used for secondary verification of the presence of publication bias, which showed *P* = 0.933 (>0.05), indicating that there is no publication bias in this study (Supplementary Table 2).

**Figure 2:**
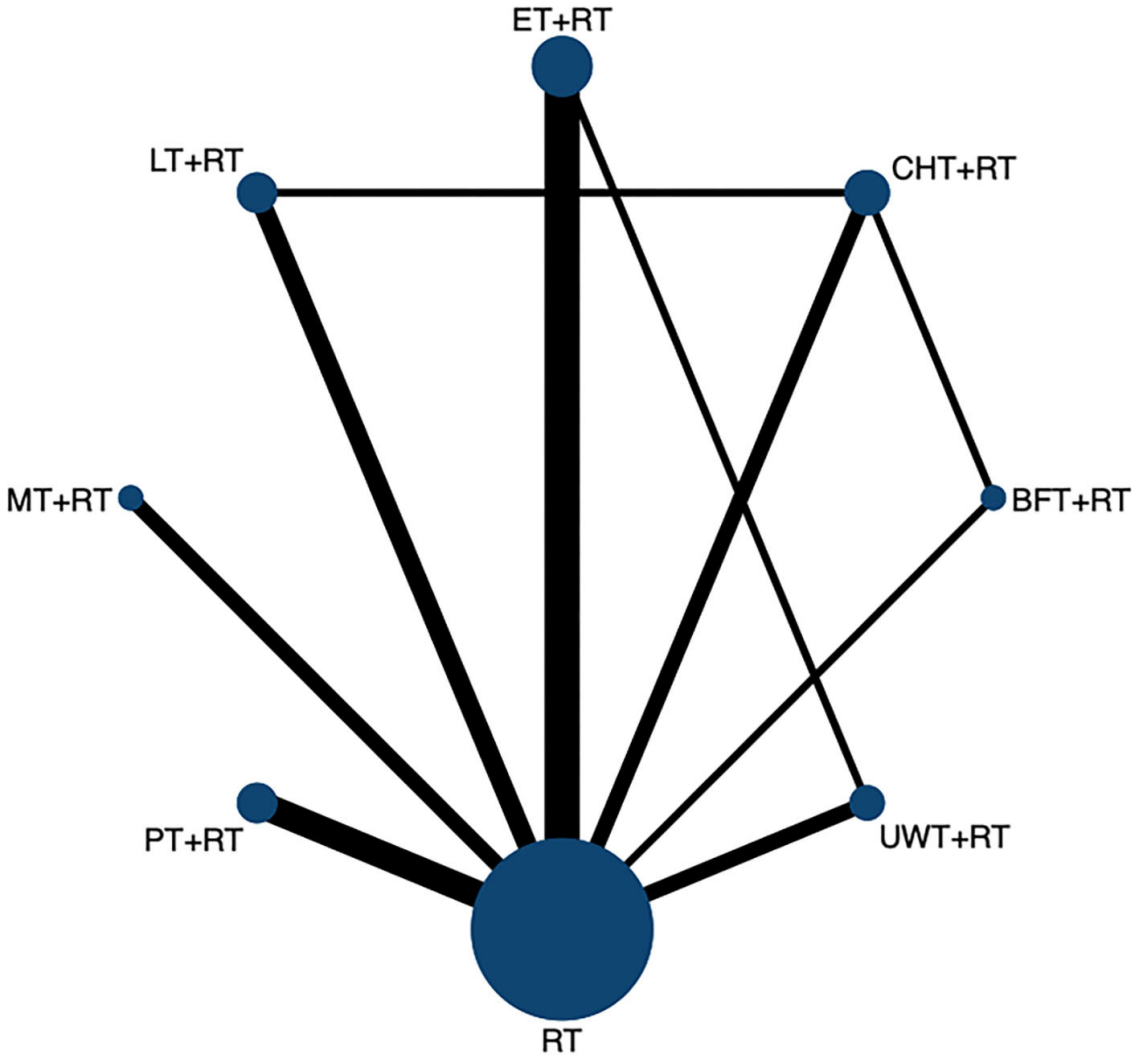
The network evidence graph for FMA-UE. RT, rehabilitation training; ET, electrotherapy; LT, light therapy; UWT, ultrasonic wave therapy; CHT, conduction heat therapy; PT, pressure therapy; MT, magnetic therapy; BFT, biofeedback therapy.

We constructed a visual network geometry showing all the main evidence of the interventions. Each node represents one intervention, and its size depends on the number of patients directly studied. As shown in Figure 2, the most common intervention method was ET + RT with nine groups studied (*n* = 313), followed by PT + RT (*n* = 259) involving seven groups, CHT + RT (*n* = 292) and UWT + RT (*n* = 174) involving six groups, and LT + RT (*n* = 265) involving five groups. Two other interventions [BFT + MT (*n* = 135) and MT + RT (*n* = 132)] involved four groups.

**Figure 3:**
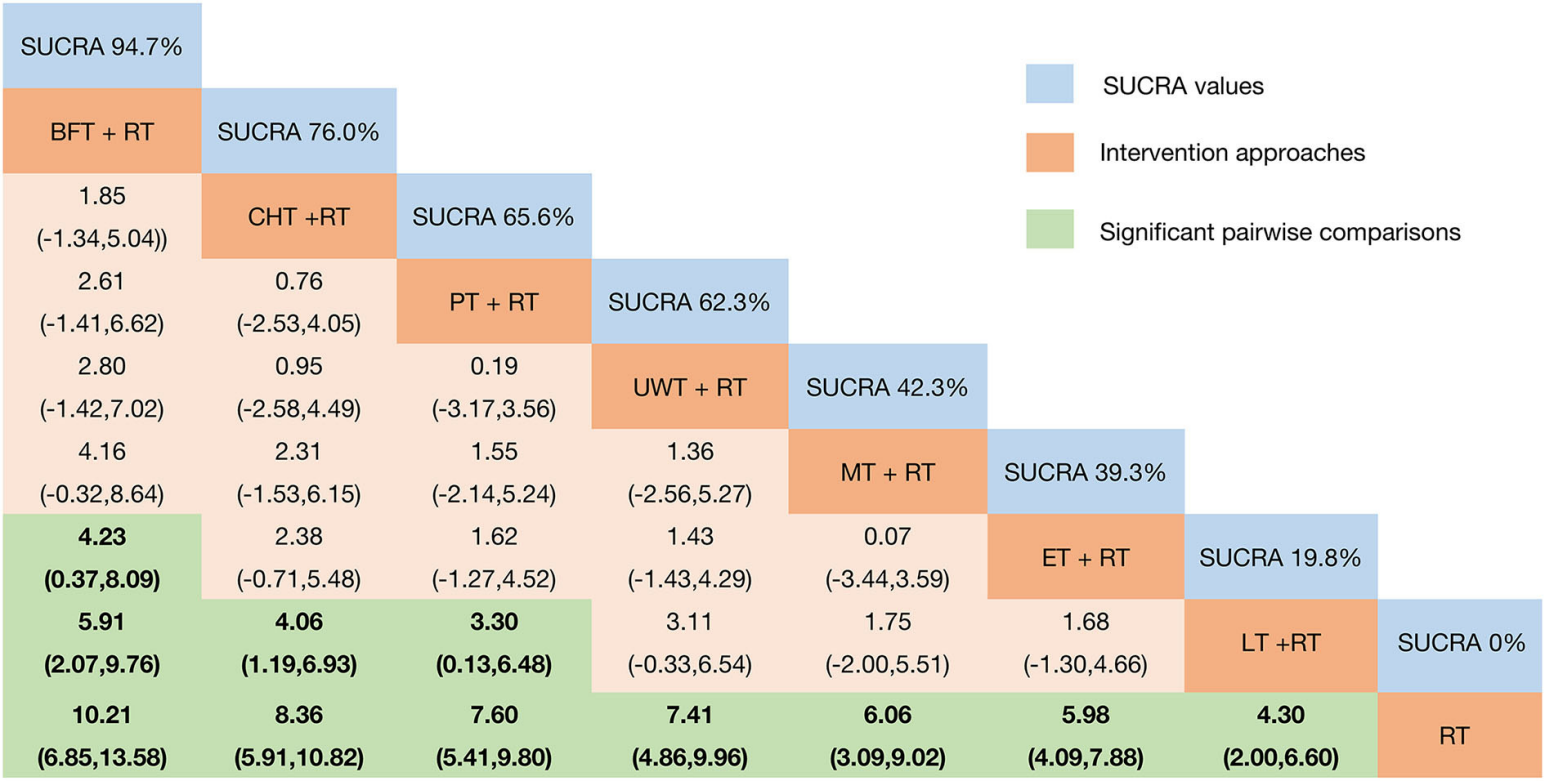
Relative effect sizes of FMA-UE efficacy after the intervention according to network meta-analysis. Treatments were ranked in order of their likelihood of being the best treatment. The numbers in the blue boxes are SUCRA values, representing the rank of the treatments. Meaningful pairwise comparisons are highlighted in green and bold. RT, rehabilitation training; ET, electrotherapy; LT, light therapy; UWT, ultrasonic wave therapy; CHT, conduction heat therapy; PT, pressure therapy; MT, magnetic therapy; BFT, biofeedback therapy.

In terms of the outcome of FMA-UE, the efficacy of various physical factor therapies (PFT) combined with rehabilitation training (RT) post-intervention is shown in Figure 3. BFT + RT [MD = 10.21 95%CrI (6.85, 13.58)]; CHT + RT [MD = 8.36 95%CrI (5.91, 10.82)]; PT + RT [MD = 7.60 95%CrI (5.41, 9.80)]; UWT + RT [MD = 7.41 95%CrI (4.86, 9.96)]; MT + RT [MD = 6.06 95%CrI (3.09, 9.02)]; ET + RT [MD = 5.98 95%CrI (4.09, 7.88)]; and LT + RT [MD = 4.30 95%CrI (2.00, 6.60)] efficacy were all statistically significant and significantly superior to the control group. BFT + RT [MD = 5.91 95%CrI (2.07, 9.76)]; CHT + RT [MD = 4.06 95%CrI (1.19, 6.93)]; and PT + RT [MD = 3.30 95%CrI (0.13, 6.48)] were all superior to LT + RT. Meanwhile, BFT + RT [MD = 4.23 95%CrI (0.37, 8.09)] also outperformed ET + RT.

We plotted SUCRA lines to rank each intervention category (Figure 3 and Supplementary Figure 5) and compared them with other interventions. BFT+RT (SUCRA = 94.7%) had the highest probability of improving upper extremity motor function in patients with post-stroke SHS, followed by two equally remarkable interventions CHT+RT (SUCRA = 76.0%) and PT+RT (SUCRA = 65.6%), and the fourth-ranked UWT+RT (SUCRA = 62.3%). In contrast, MT+RT (SUCRA = 42.3%), ET+RT (SUCRA = 39.3%), and LT+RT (SUCRA = 19.8%) had relatively low probabilities, while the probability of RT (SUCRA = 0%) was the lowest. The existence of inconsistencies between direct and indirect evidence was assessed by the “nodal split” method. The results (Supplementary Figure 6) showed that there are no significant inconsistencies in each branch of the entire network (*P* > *0.05*) [CHT + RT vs. RT (*P* = 0.566); LT + RT vs. RT (*P* = 0.123); UWT + RT vs. RT (*P* = 0.496); ET + RT vs. RT (*P* = 0.498); BFT + RT vs. RT (*P* = 0.321); LT + RT vs. CHT + RT (*P* = 0.123); BFT + RT vs. CHT + RT (*P* = 0.325); and ET + RT vs. UWT + RT (*P* = 0.50)]. Thus, we obtained a valid comparison of the above-mentioned different physical therapy interventions to improve the function of the upper limb of SHS after stroke.

#### Secondary outcome: VAS

The *I*^2^ values indicated that our preliminary meta-analysis showed high heterogeneity in VAS scores across all included studies (*I*^2^ = 82.2%, *P* = 0.000). Comparison-adjusted funnel plot suggested that the occurrence of publication bias depends on several scattered points that are asymmetrically distributed below and outside the inverted funnel plot (Supplementary Figure 7). In addition, Egger's test confirmed this result (*P* = 0.011) (Supplementary Table 3).

**Figure 4:**
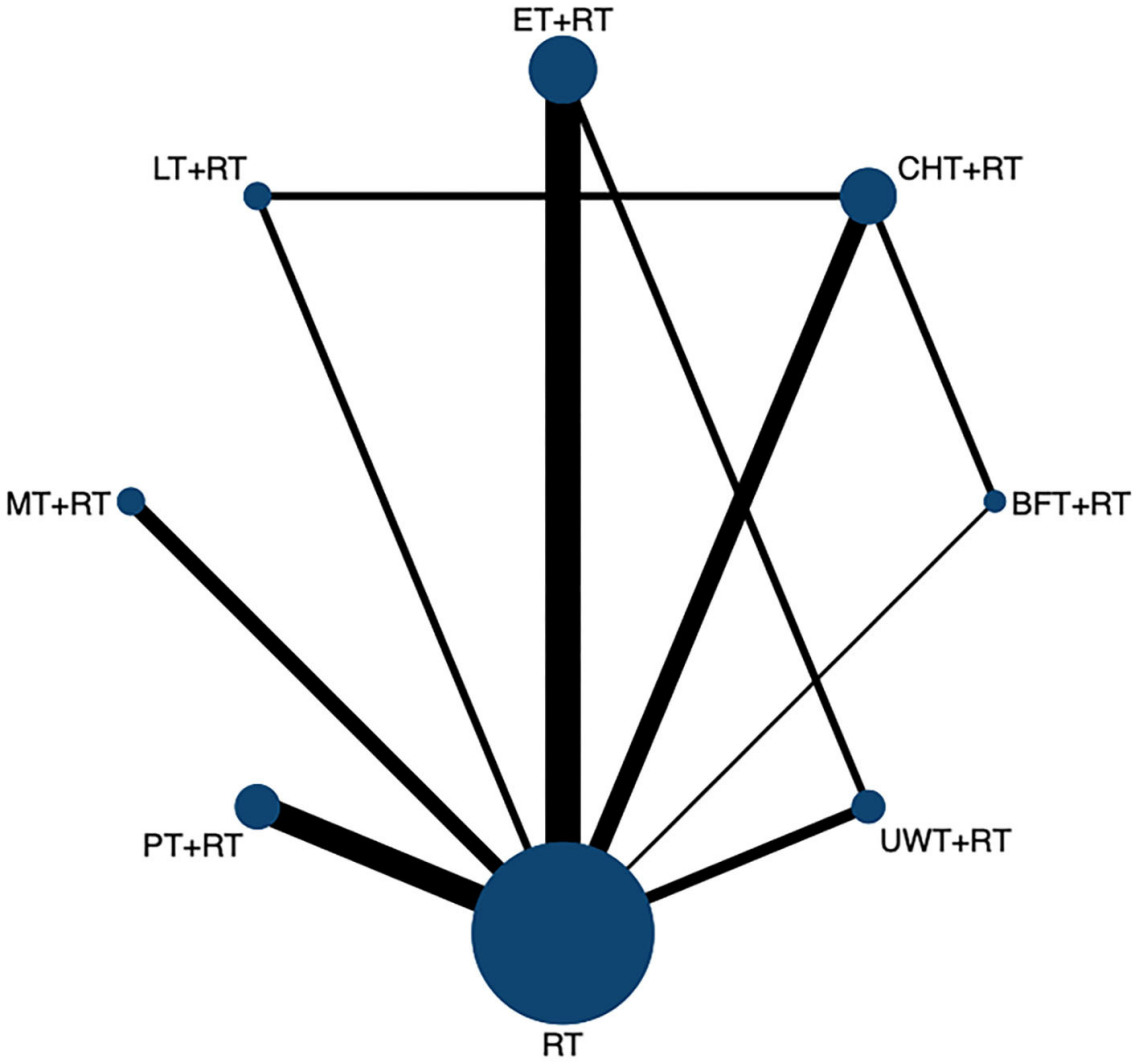
The network evidence graph for VAS. RT, rehabilitation training; ET, electrotherapy; LT, light therapy; UWT, ultrasonic wave therapy; CHT, conduction heat therapy; PT, pressure therapy; MT, magnetic therapy; BFT, biofeedback therapy.

The network diagram is shown in Figure 4, including seven interventions and four control groups. ET + RT was the most frequent intervention and investigated in 9 arms (*n* = 303), followed by the most common intervention of CHT + RT (*n* = 352) and PT + RT (*n* = 244) involving 7 arms; UWT + RT (*n* = 144) involving 5 arms; MT + RT (*n* = 132) involving 4 arms; BFT + RT (*n* = 105) involving 3 arms; and LT + RT (*n* = 120) was the least involving only 2 arms.

**Figure 5:**
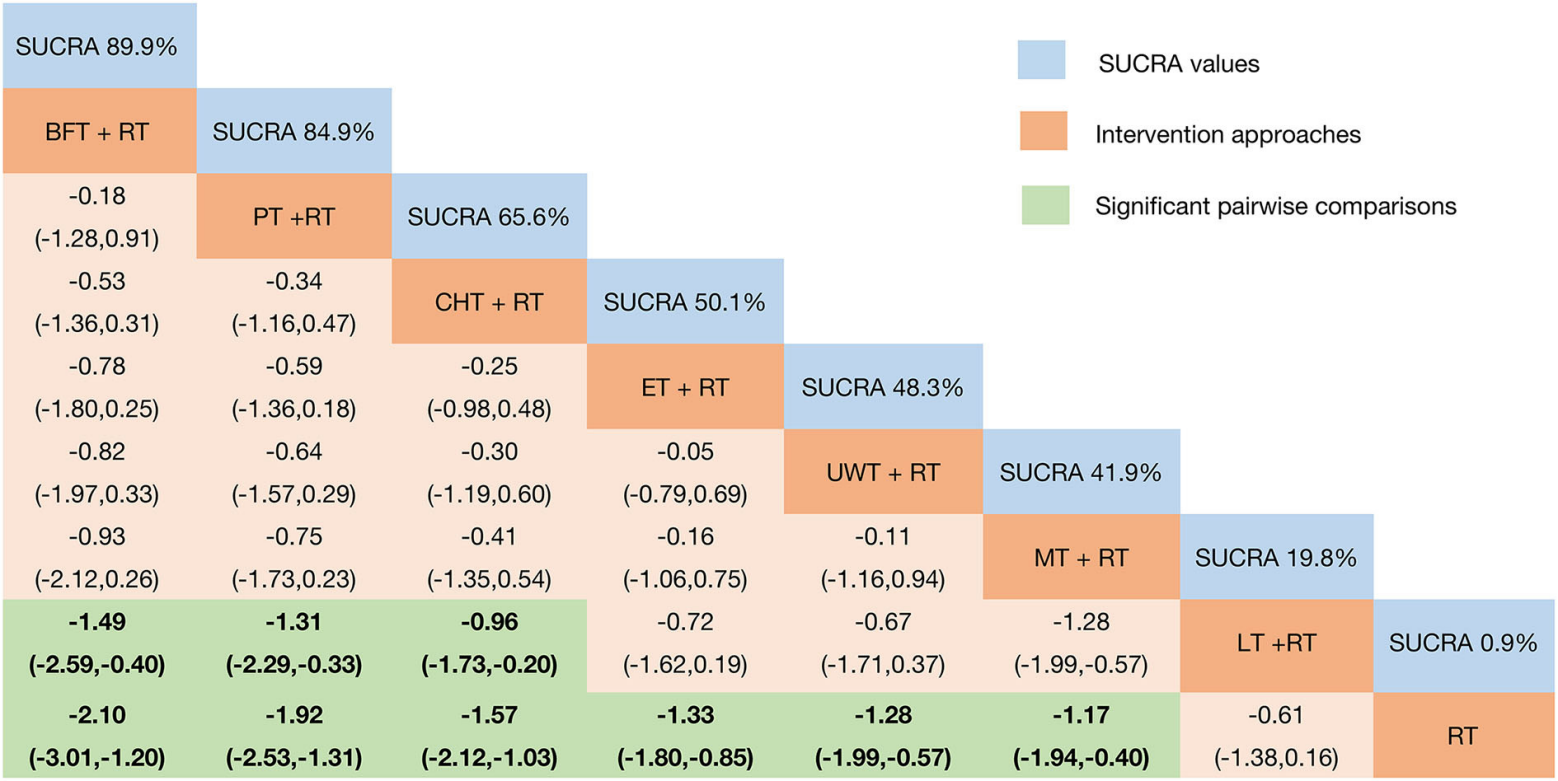
Relative effect sizes of VAS efficacy after the intervention according to network meta-analysis. Treatments were ranked in order of their likelihood of being the best treatment. The numbers in the blue boxes are SUCRA values, representing the rank of the treatments. Meaningful pairwise comparisons are highlighted in green and bold. RT, rehabilitation training; ET, electrotherapy; LT, light therapy; UWT, ultrasonic wave therapy; CHT, conduction heat therapy; PT, pressure therapy; MT, magnetic therapy; BFT, biofeedback therapy.

The clinical efficacy of VAS pain relief results showed (Figure 5) that when compared with the control group, except for LT + RT, the other interventions showed better efficacy: BFT + RT [MD = −2.10 95%CrI (−3.01, −1.20)]; PT + RT [MD = −1.92 95%CrI (−2.53, −1.31)]; CHT + RT [MD = −1.57 95%CrI (−2.12, −1.03)]; ET + RT [MD = −1.33 95%CrI (−1.80, −0.85)]; UWT + RT [MD = −1.28 95%CrI (−1.99, −0.57)]; and MT + RT [MD = −1.94 95%CrI (−1.94, −0.40)]. In addition, BFT + RT [MD = −1.49 95%CrI (−2.59, −0.40)]; PT + RT [MD = −1.31 95%CrI (−2.29, −0.33)]; and CHT + RT [MD = −0.96 95%CrI (−1.73, −0.20)] were also significantly superior than LT + RT.

Plotting the SUCRA line to rank each intervention's efficacy in pain relief (Figure 5 and Supplementary Figure 8) showed that BFT + RT (SUCRA = 89.9%) obtained the best probability compared to the other seven interventions. However, PT + RT (SUCRA = 84.9%) and CHT + RT (SUCRA = 65.8%) also got a remarkable ranking among them, followed by ET + RT (SUCRA = 50.1%); UWT + RT (SUCRA = 48.3%); and MT + RT (SUCRA = 41.9%). LT + RT (SUCRA = 18.3%) and RT (SUCRA = 0.9%) ranked last. The node-splitting model results showed (Supplementary Figure 9) no significant inconsistency between the direct and indirect evidence (*P* > 0.05), so the current evidence is reliable.

## Discussion

Existing RCTs have only analyzed the relative effectiveness of individual physical therapy interventions in terms of their respective efficacy in patients with post-stroke SHS. At the same time, traditional meta-analyses have only been used to assess the effectiveness of a particular intervention. It all lacks comprehensive comparative analyses between studies, but the NMA overcomes this limitation. Network meta-analysis integrates at least two or more physical interventions by performing direct and indirect cross-comparisons with the help of techniques that adjust indirect comparisons while assessing their effectiveness and performing relative ranking on all physical therapy interventions included (75). To the best of our knowledge, this is the first study to use the NMA approach to compare the efficacy of different physical therapy for patients with SHS after stroke. This complex integrated approach is superior to most previous studies, and it can be used as an evidence-based clinical guideline to provide reference evidence for the selection of optimal protocols for the future clinical treatment of SHS.

Post-stroke SHS is a complex disease that threatens the recovery of patients with stroke, and it is essential to identify effective treatment strategies. Although rehabilitation is effective in treating SHS, pain is the primary reason that prevents patients from receiving SHS treatment. In addition, it leads to resistance psychology in some patients, affecting their treatment outcomes (20, 83). Evidence suggests that combining two or more therapies may be more effective than rehabilitation alone in improving the post-stroke SHS symptoms of patients. Physical therapy, in particular, has shown superior performance in reducing pain and improving motor function as the first-line treatment choice for this disease (17, 84–86). Among them, biofeedback therapy (BFT) with EMG biofeedback as the primary intervention combined with rehabilitation training (RT) may offer the potential for the treatment of SHS. In this study, both FMA and VAS results showed that BFT + RT [(MD = 10.21 95%CrI (6.85, 13.58), (SUCRA = 94.7%); (MD = −2.10 95%CrI (−3.01, −1.20), (SUCRA = 89.9%)] is the best treatment strategy to improve upper limb motor function and reduce pain in patients with SHS.

Electromyographic biofeedback (EMG-BF) therapy, a branch of biofeedback therapy (BFT), combines biofeedback techniques with electrical stimulation to promote the reconstruction of undamaged nerve cells and the development of new neural networks after stroke (84). By amplifying the bioelectrical activity of muscle tissue, which the patient is unaware of under normal circumstances, and processing the signal, the signal is fed back to the human body as intuitive visual and auditory signals and further fed back to the brain center. The brain control center regulates muscle contraction and diastole intensity based on the feedback signal and receives active rehabilitation training to achieve the goal of training and treatment (85). According to the results of the meta-analysis of this study, we found that there are statistical differences in the comparison of the efficacy of BFT + RT and electrical stimulation therapy (ET) combined with RT [MD = 4.23 95%CrI (0.37, 8.09)] in improving limb motor function. The results also confirmed the advantages of EMGBF treatment. It overturns the traditional notion that autonomic nerves cannot be controlled arbitrarily and allows patients to dynamically access electromyographic physiological information at the site of information collection, enabling them to learn to consciously regulate their psychophysiological activity to treat somatic disorders (86). It has been demonstrated (87) that EMG-BF provides an additional benefit for the recovery of limb function in patients with stroke when combined with conventional rehabilitation. Moreover, its efficacy is undoubtedly substantial. In addition to promoting the recovery of neurological deficits after stroke, it also helps patients overcome pain-induced resistance to training and motivates them to participate actively in rehabilitation (88, 89). Related studies (43) found that using surface EMG-BF to treat stroke patients with SHS can improve patients' ability to control and regulate random movements significantly. Meanwhile, it also stimulates their desire to train, which transforms passive rehabilitation into active rehabilitation, and improves patients' compliance with training, leading to improved patient outcomes.

In contrast, the potential mechanism of EMG biofeedback in pain relief remains unclear. Related studies found (90) that through the “stimulation-feedback” mode, EMGBF is capable of converting subtle EMG signals into visual stimuli, thereby motivating patients to engage in active exercises of the core muscles of the affected shoulder to stabilize the shoulder joint and alleviate pain. The problem is that when hemiplegic shoulder pain is caused by the interaction of multiple etiologies, a single therapy may not be able to achieve the desired level of pain relief (91, 92). However, our study draws the opposite conclusion, which may be related to our combing EMG biofeedback with rehabilitation training and thus improved efficacy; or it may be associated with the lack of direct evidence between interventions. Speculation on this contradictory view still needs to be validated by more extensive RCTs of the combined treatment with myoelectric biofeedback on shoulder pain in the future.

Also noteworthy is that CHT + RT and PT + RT rank relatively high among all interventions and can be used adjunctively for post-stroke SHS. CHT mainly consists of paraffin wax therapy and moist heat compress therapy. Wax therapy, as a particular conductive medium, uses this principle of warming to conduct heat through the skin to deep tissues, accelerating tissue repair, promoting cellular metabolism, reducing the tension of tissue fibers, and increasing their elasticity. It thereby facilitates muscle strength recovery and enhances joint mobility. At the same time, the warming effect can reduce the excitability of the nerve, improve blood circulation, and finally, reduce inflammatory edema, and accelerate the removal of pain-causing mediators. Furthermore, when the wax is cooled, its fixed condition exerts a local oppressive impact on the body's tissues, aiding in the eradication of swelling and having a better effect on the relaxation of the affected joint ligaments, muscles, and tendons (93). Clinical studies have demonstrated that functional training of the upper extremity soon following the wax therapy can help patients better participate in the training and complete their rehabilitation activities better (46, 94). Wet heat compress therapy, also referred to as Chinese herbal medicine moist heat compress therapy, is often combined with Chinese herbal medicine. Using the combined effects of herbal efficacy and physical thermal effect to select herbs that reduce inflammation, alleviate pain, and relax tendons have many advantages. It can dilate the local blood vessels and open pores, which deepens the drug penetration and gives full play to its effect. Consequently, it improves the time effect of pain symptom relief and facilitated the metabolism of inflammation and edema. Additionally, it significantly increased blood flow to the affected limb's tissues, lowered muscle and ligament tension, and enhanced the flexibility of joints and limb movements, thus improving therapeutic results (95, 96). They have limitations, however, and should be used with caution in patients who have the bleeding tendency in clinical, local sensory abnormality, or wax allergy (86).

Pressure therapy (PT) mainly refers to interstitial pneumatic therapy. With the use of an air pump, the multi-chambered balloon is inflated uniformly and decompressed in an orderly manner, providing centripetal compression from distal to proximal segments of the limb and improving arterial perfusion. It effectively improves arterial blood circulation in the affected limb, thereby eliminating edema and improving peripheral vascular function (66, 97). However, given that pneumatic therapy inflation and deflation are neither based on blood flow blockage and recovery pressure nor does it take into account the influence of the patient's upper limb circumference on the pneumatic therapy pressure, and that patients with stroke frequently have sensory impairment of the affected limb, judging the pneumatic therapy pressure based on the patient's subjective sensation alone lacks scientific validity and may cause adverse effects (98). Consequently, pneumatic therapy has been used in relatively few RCTs to treat this disease alone, mainly as adjunctive therapy after rehabilitation training to provide muscle relaxation and pain relief. As previously stated, our findings also found that PT + RT is more effective in relieving pain (SUCRA = 84.9%) than improving limb motor function (SUCRA = 65.6%).

In addition, studies showed (99, 100) that ultrasonic wave therapy (UWT) also has mechanical and thermal physical effects. By directly acting on local subcutaneous tissue, the ultrasound emitted from outside the body is concentrated in the deep surface of the tissue and produces a high-energy point, which causes the lesion tissue to absorb energy in a short period of time and rapidly heat up, and produces physical and chemical effects. Ultimately, it promotes local blood circulation, accelerates the absorption of inflammatory factors, reduces the excitability of sensory nerves, and cures pain. However, based on the evidence of this study, UWT did not present a prominent advantage, especially in terms of pain relief (SUCRA = 48.3%). This may be related to the lack of significant differences between various physical therapy interventions and may also be influenced by the number of relevant RCTs available for inclusion, resulting in a lack of more direct comparative evidence. Similarly, the relatively weak ranking of magnetic therapy (MT) with transcranial magnetic stimulation as the main intervention may be explained by the relative paucity of studies on the clinical use of magnetic therapy for SHS compared to others (*n* = 132). Nevertheless, again, this needs further confirmation.

More noteworthy is that, according to our pooled meta-analysis, no statistical difference was observed in pain relief between the light therapy combined with rehabilitation training (LT + RT) group and the control group. Also, based on the SUCRA values, the top three ranked physiotherapies (BFT + RT, CHT + RT, PT + RT) are all statistical differences compared to LT + RT. Generally, this finding is consistent with recent studies (101), indicating that phototherapy has a weak immediate analgesic effect and that its long-term effectiveness is mainly determined by its ability to repair tissues. Therefore, it is commonly used in the adjunctive treatment of pain diseases. On the contrary, the possible differences in the methodological design of different current studies result from the continuous advancement of medicine and the emergence of new high-energy lasers and helium-neon lasers. However, due to the setting of inclusion criteria and other technical limitations, we failed to explore this aspect in depth. This remains to be analyzed in the future by further collecting more direct evidence.

## Limitations

However, our study has some limitations as well. First, our study aims to make comparisons from a macroscopic perspective, thus ignoring the refined specific interventions such as transcutaneous electrical nerve stimulation, intermediate frequency electrotherapy, and hyperbaric oxygen or confounding factors such as different frequencies and different intervention durations. Second, the included studies which were all from China lack ethnic diversity, which may result in the limited generalizability of the findings. Finally, significant differences in sample sizes between physical therapy interventions may also have contributed to imprecise analyses. Compared with the overall sample size (*n* = 3379), the sample sizes of BFT + RT (*n* = 135) and MT + RT (*n* = 132) are relatively small.

## Conclusion

Based on the findings of our NMA study, EMG biofeedback therapy combined with rehabilitation training (BFT + RT) is the most effective physiotherapy option for improving upper extremity motor function and relieving pain in patients with the post-stroke SHS, followed by CHT + RT and PT + RT. However, given the macroscopic nature of this study and the lack of direct comparative evidence between multiple countries and centers, future studies need to conduct related randomized controlled trials on more physiotherapy interventions. In addition, it helps to conduct more relevant and refined meta-analyses successfully.

## Data availability statement

The original contributions presented in the study are included in the article/Supplementary material, further inquiries can be directed to the corresponding author.

## Author contributions

SF conceived the study and wrote the manuscript. MT, GH, and JW participated in the extraction and analysis of the data. The study was critically supervised, evaluated, and validated by LG, SH, and DL. All of the authors worked on the article and agreed with the version that was sent in.

## Supplementary material

Supplementary Figure 1 Model convergence and density plots.

Supplementary Figure 2 Quality assessment percentage graph.

Supplementary Figure 3 Summary chart of quality assessment.

Supplementary Figure 4 FMA-UE funnel plot.

Supplementary Figure 5 Probability ranking results of FMA-UE of different interventions.

Supplementary Figure 6 Node-splitting diagram of FMA-UE.

Supplementary Figure 7 VAS funnel plot.

Supplementary Figure 8 Probability ranking results of VAS of different interventions.

Supplementary Figure 9 Node-splitting diagram of VAS.

Supplementary Table 1 The search strategy for PubMed.

Supplementary Table 2 Egger's test for FMA-UE.

Supplementary Table 3 Egger's test for VAS.
