# Supplementary figures and images for: EMG biofeedback combined with rehabilitation training may be the best physical therapy for improving upper limb motor function and relieving pain in patients with the post-stroke shoulder-hand syndrome: A Bayesian network meta-analysis
Source: Front Neurol. 2023 Jan 10;13:1056156. doi: 10.3389/fneur.2022.1056156 (PMC9873378; doi:10.3389/fneur.2022.1056156)

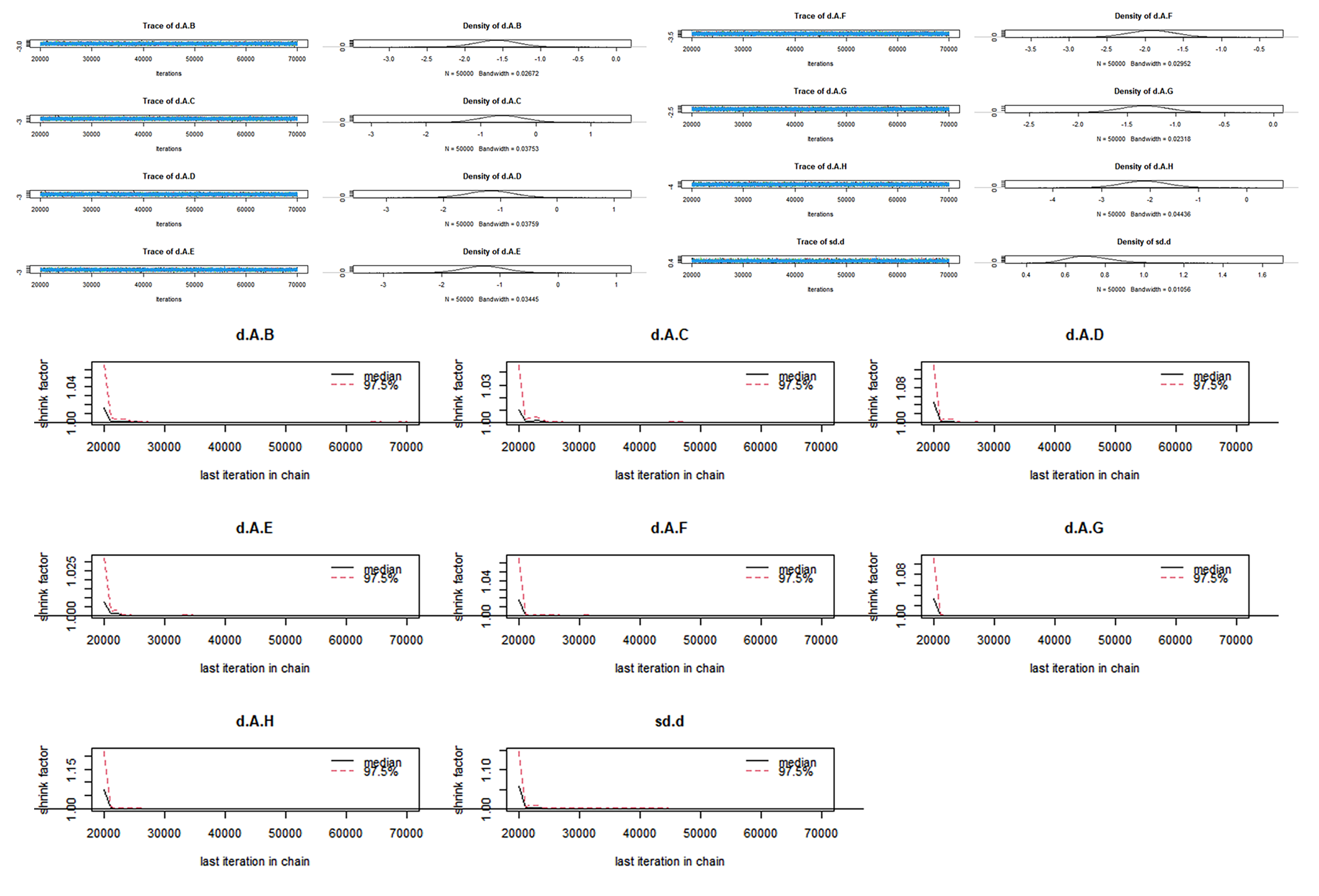

Supplement: Supplementary Figure 1 — Model convergence and density plots. [file Image_1.TIF]

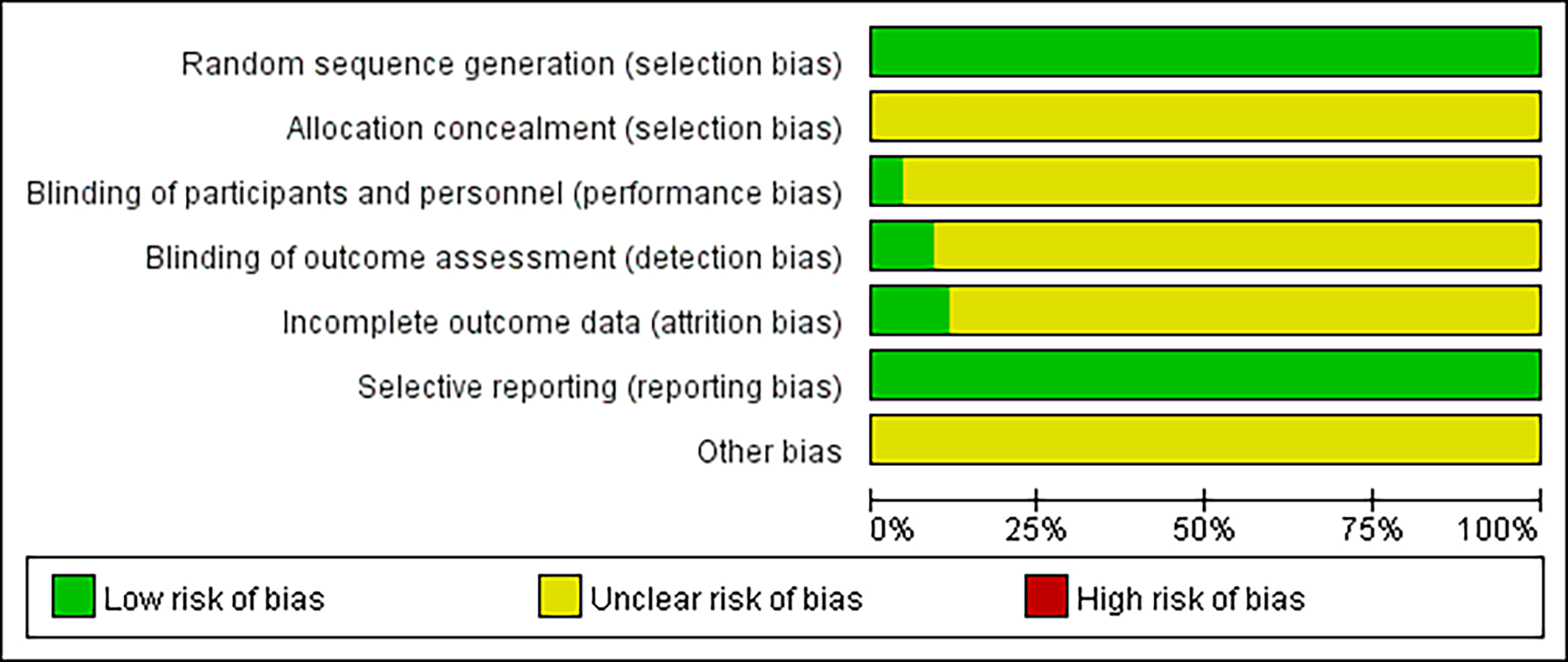

Supplement: Supplementary Figure 2 — Quality assessment percentage graph. [file Image_2.TIF]

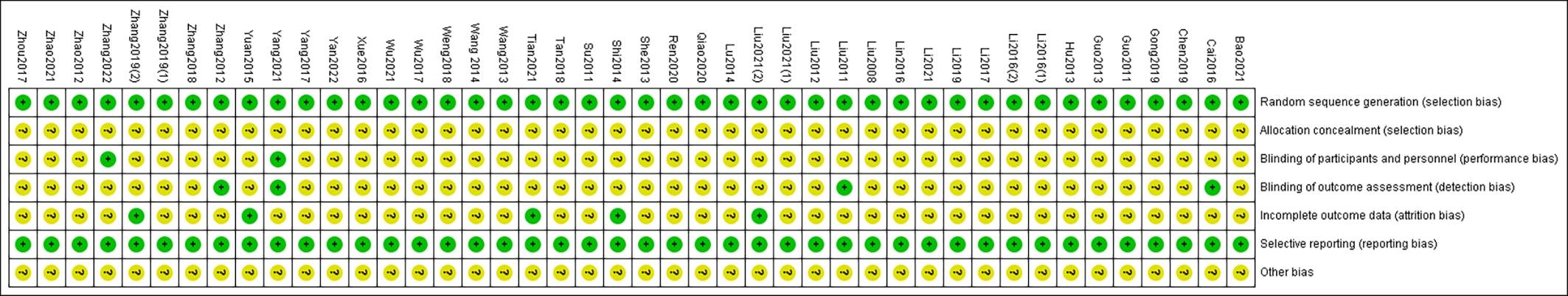

Supplement: Supplementary Figure 3 — Summary chart of quality assessment. [file Image_3.TIF]

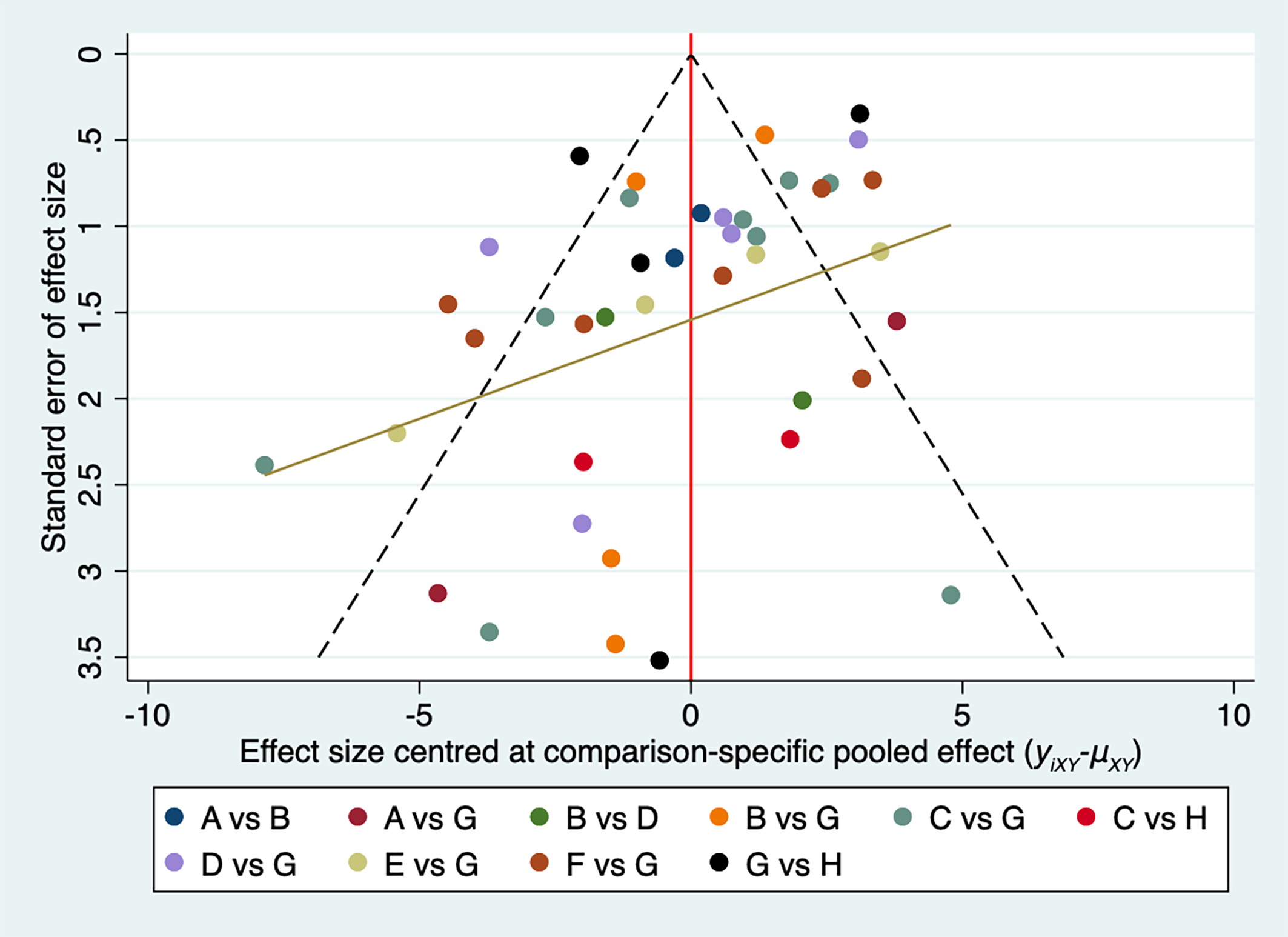

Supplement: Supplementary Figure 4 — FMA-UE funnel plot. [file Image_4.TIF]

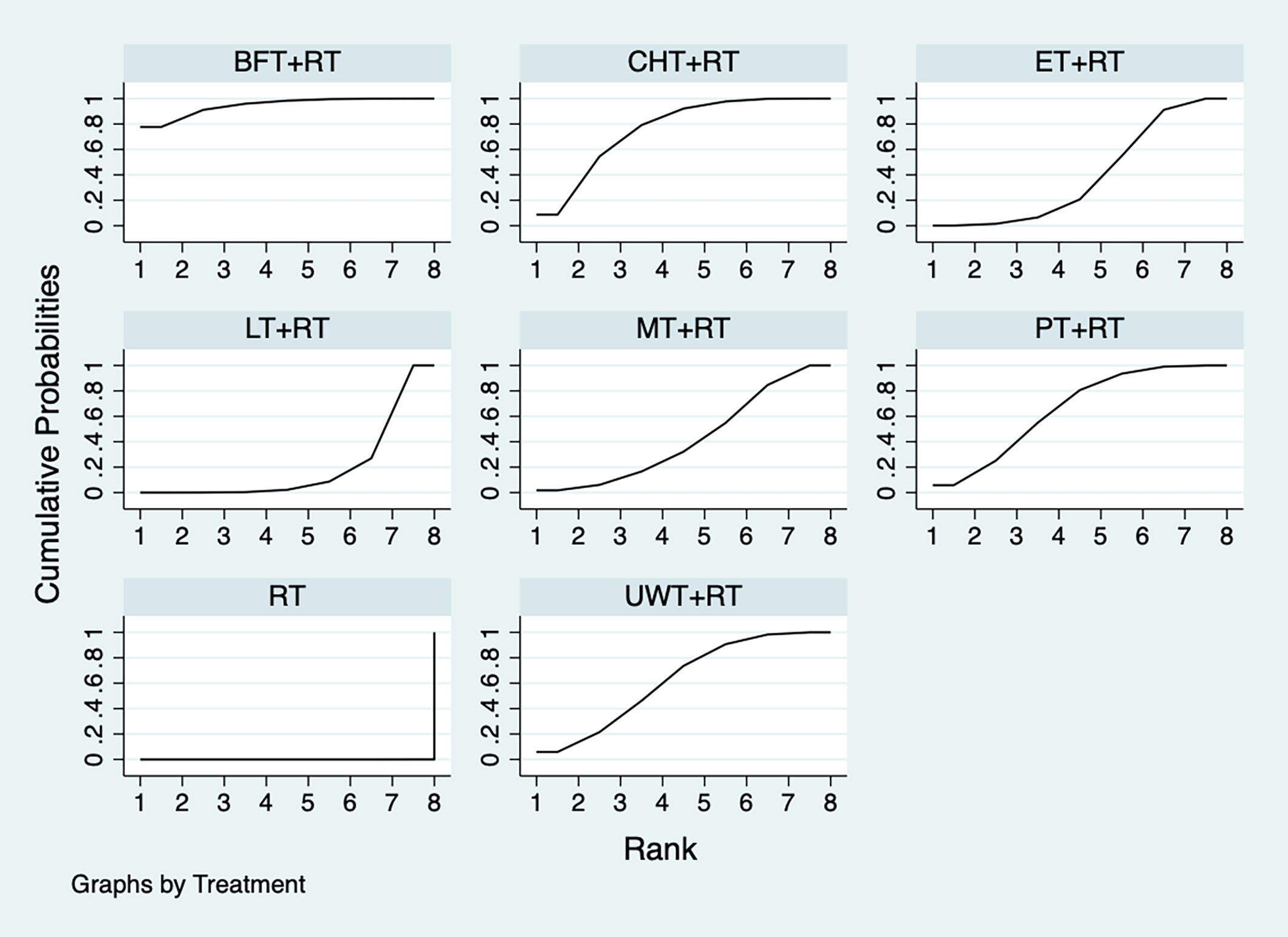

Supplement: Supplementary Figure 5 — Probability ranking results of FMA-UE of different interventions. [file Image_5.TIF]

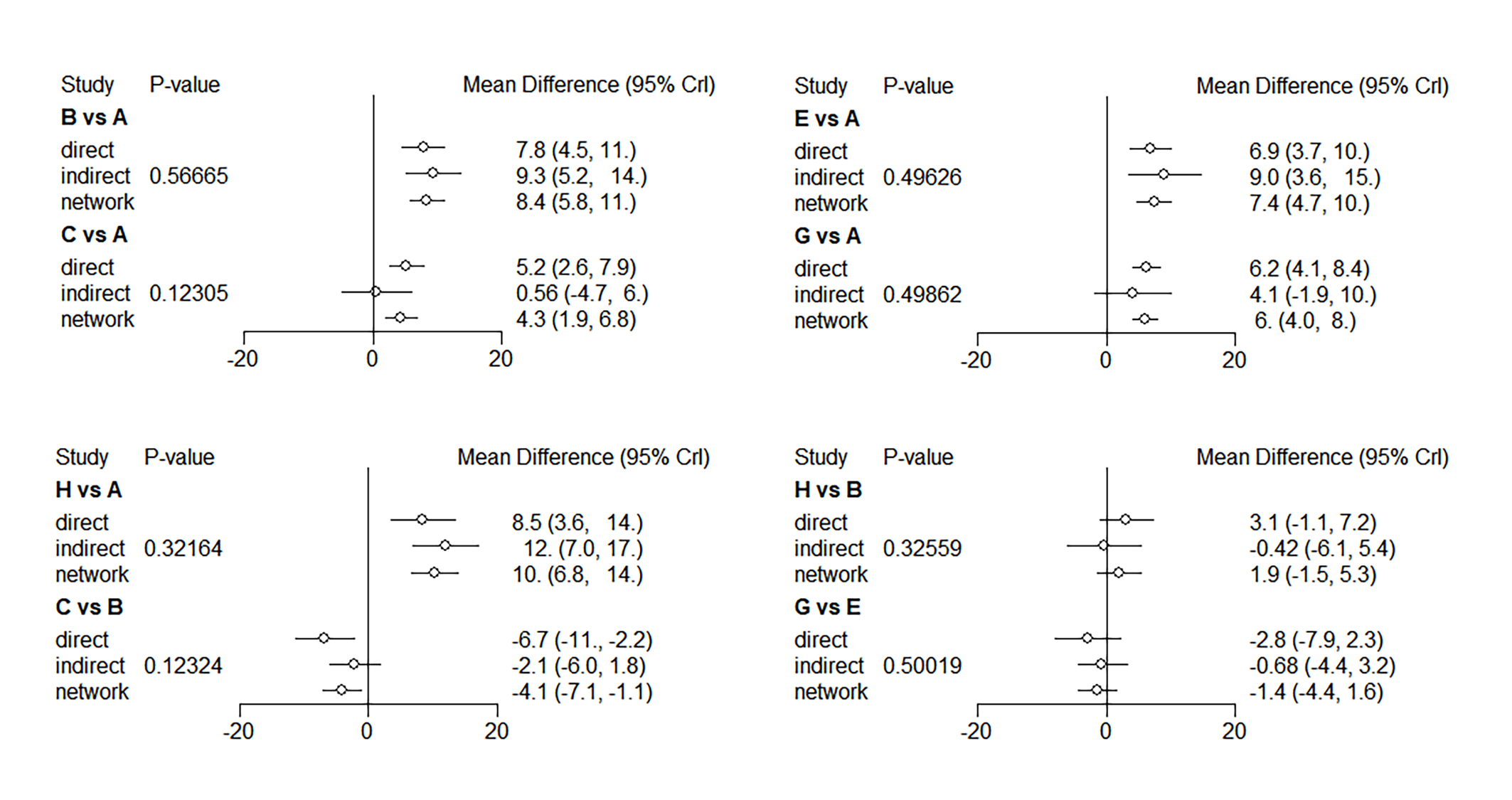

Supplement: Supplementary Figure 6 — Node-splitting diagram of FMA-UE. [file Image_6.TIF]

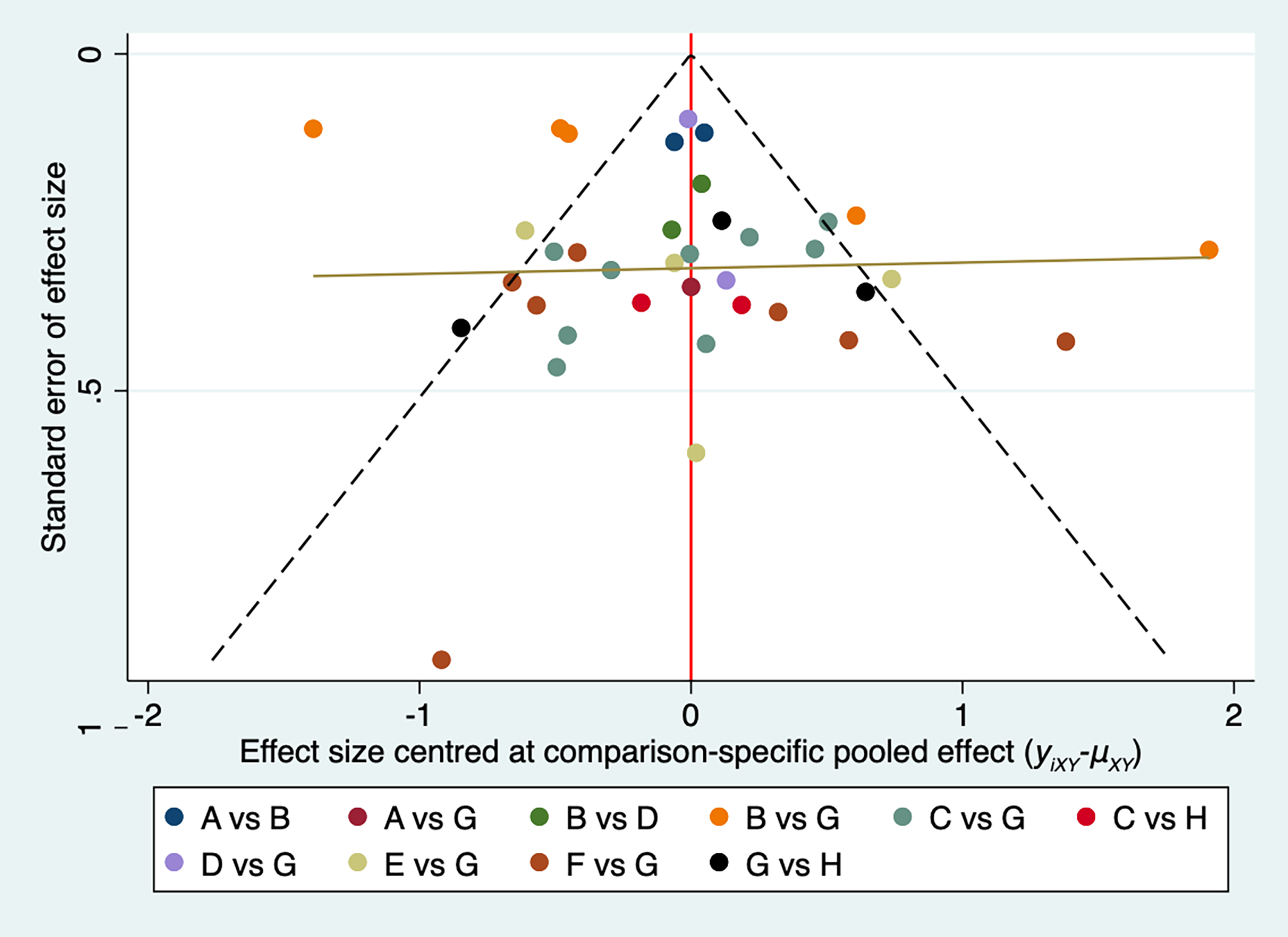

Supplement: Supplementary Figure 7 — VAS funnel plot. [file Image_7.TIF]

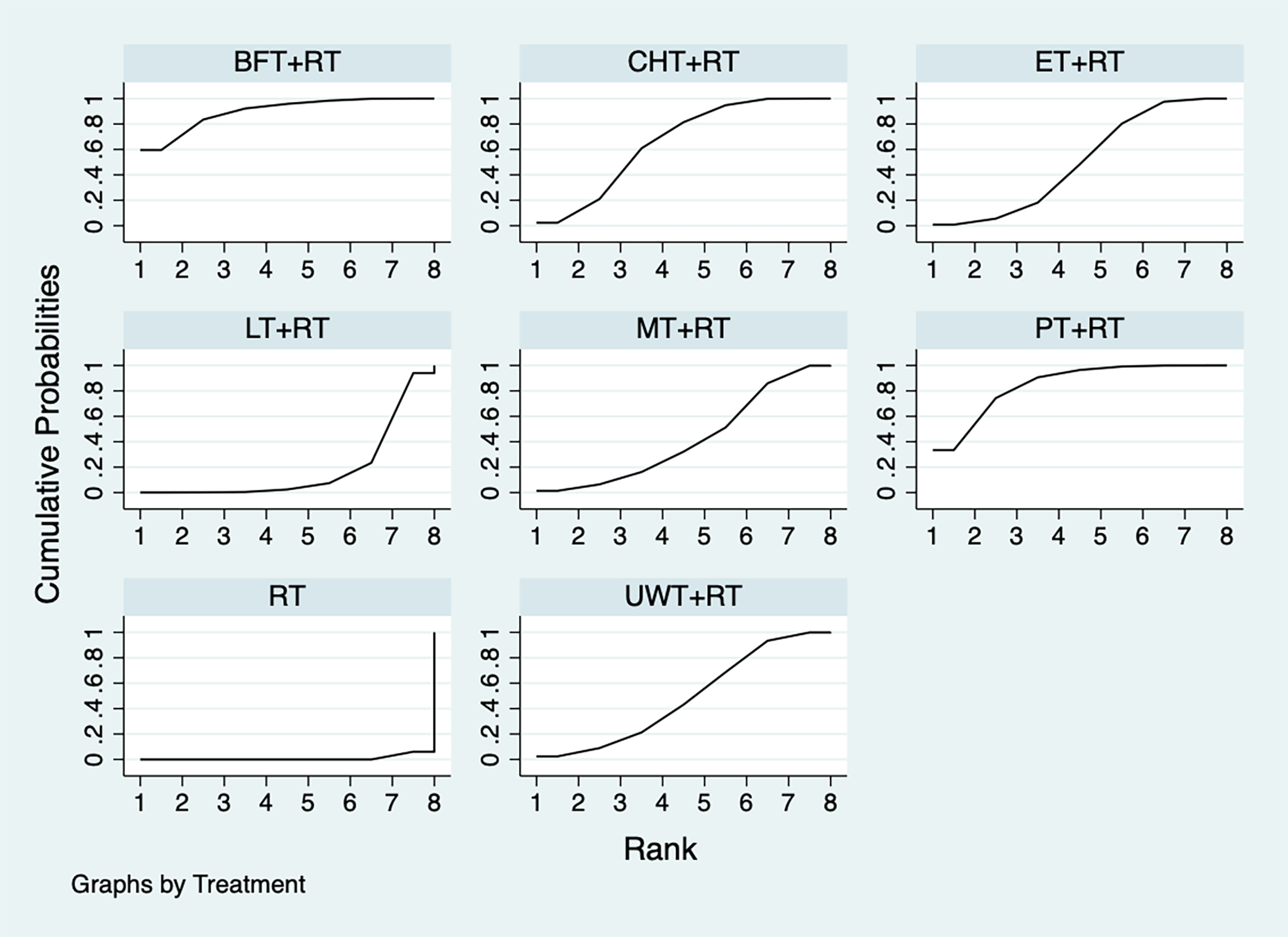

Supplement: Supplementary Figure 8 — Probability ranking results of VAS of different interventions. [file Image_8.TIF]

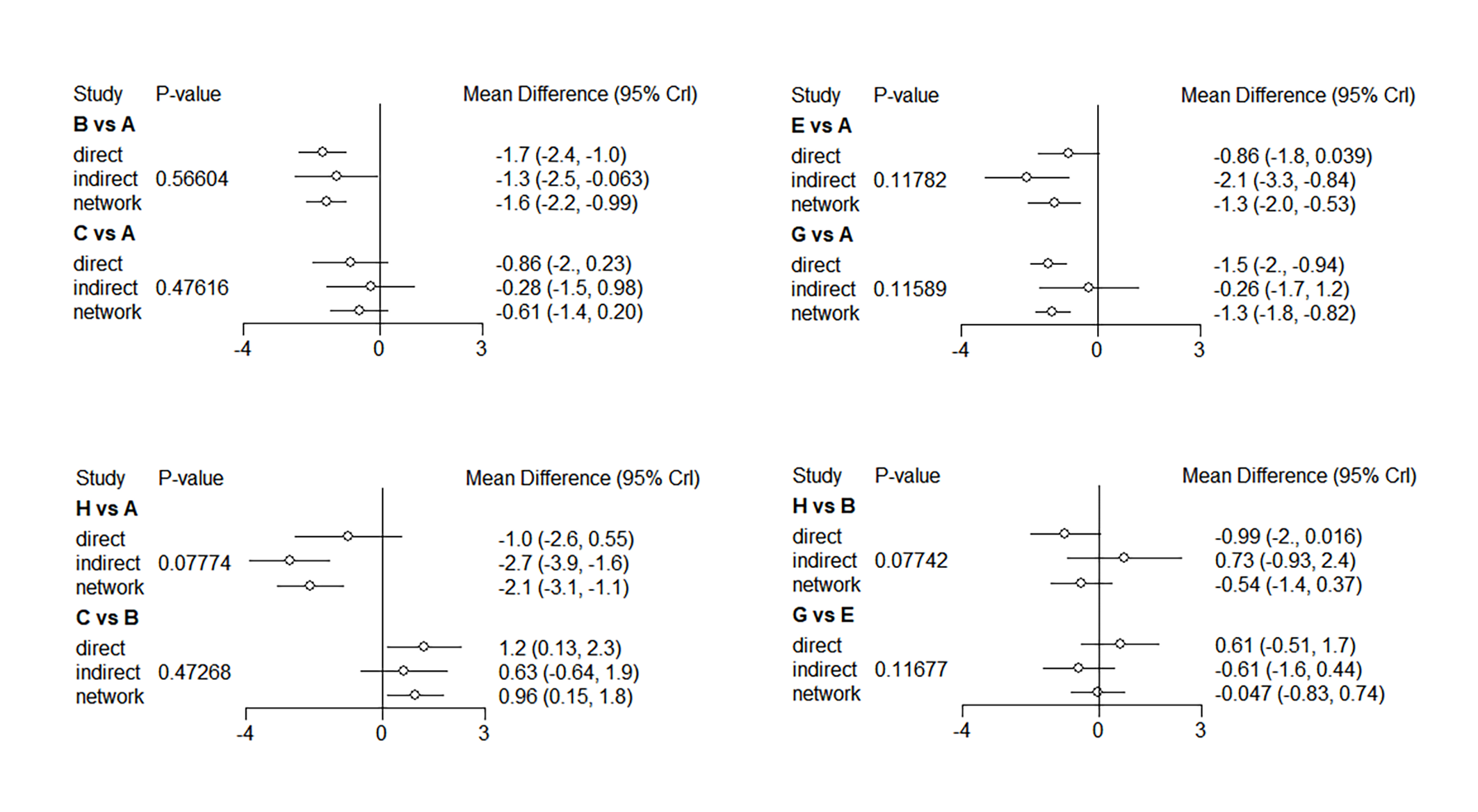

Supplement: Supplementary Figure 9 — Node-splitting diagram of VAS. [file Image_9.TIF]
