# Supplementary material for: EMG biofeedback combined with rehabilitation training may be the best physical therapy for improving upper limb motor function and relieving pain in patients with the post-stroke shoulder-hand syndrome: A Bayesian network meta-analysis
Source: Front Neurol. 2023 Jan 10;13:1056156. doi: 10.3389/fneur.2022.1056156 (PMC9873378; doi:10.3389/fneur.2022.1056156)
Supplement: Supplementary Table 1 — The search strategy for PubMed. [file Table_1.DOCX]

Supplementary Material 1. The search strategy for PubMed.

| Number | Search terms |
| --- | --- |
| #1 | “Stroke”[Mesh] OR“Strokes”[Title/Abstract] OR “Cerebrovascular Accident”[Title/Abstract] OR “Cerebrovascular Stroke”[Title/Abstract] OR “Apoplexy”[Title/Abstract] OR “Cerebral Stroke”[Title/Abstract]OR “Vascular Accidents, Brain”[Title/Abstract]OR “CAV”[Title/Abstract]OR “cerebral infarction”[Title/Abstract]OR “Ischemic Stroke”[Title/Abstract]OR “cerebral hemorrhage[Title/Abstract]OR “Hemorrhage Stroke”[Title/Abstract] |
| #2 | “Reflex Sympathetic Dystrophy”[Mesh] OR “Complex Regional Pain Syndrome, Type I”[Title/Abstract] OR “Shoulder-Hand Syndrome”[Title/Abstract] OR “Algodystrophy” [Title/Abstract] OR “Sudek's Atrophy” [Title/Abstract] |
| #3 | “Electrotherapy”[Mesh] OR “Low-frequency pulse electrical stimulation” [Title/Abstract] OR “neuromuscular electrostimulation”[Title/Abstract] OR “transcutaneous electrical nerve stimulation”[Title/Abstract] OR “ultrashort wave”[Title/Abstract] OR “Light Therapy”[Mesh] OR “Laser Therapy”[Title/Abstract] OR “infrared therapy ”[Title/Abstract] OR “Conduction heat therapy”[Title/Abstract] OR “wax therapy”[Title/Abstract] OR “wet-hot compress”[Title/Abstract] OR “Pressure therapy”[Title/Abstract] OR “air wave therapy”[Title/Abstract] OR “hyperbaric oxygen”[Title/Abstract] OR “Magnetotherapy”[Mesh] OR “Transcranial Magnetic Stimulation”[Title/Abstract] OR “Biofeedback therapy”[Mesh] OR “electromyographic biofeedback therapy”[Title/Abstract] |
| #4 | “Rehabilitation”[Mesh] OR “Rehabilitation Training” [Title/Abstract] OR “Habilitation” [Title/Abstract] |
| #5 | “Randomized Controlled Trial[Publication Type]OR “RCT randomized controlled”[Publication Type] OR “random allocation”[Title/Abstract] OR “allocation, random”[Title/Abstract] OR “randomized, controlled”[Title/Abstract] OR “clinical trial”[Title/Abstract] |
| #6 | #1 AND #2 AND #3 AND #4 AND #5 |
