# Supplementary material for: EMG biofeedback combined with rehabilitation training may be the best physical therapy for improving upper limb motor function and relieving pain in patients with the post-stroke shoulder-hand syndrome: A Bayesian network meta-analysis
Source: Front Neurol. 2023 Jan 10;13:1056156. doi: 10.3389/fneur.2022.1056156 (PMC9873378; doi:10.3389/fneur.2022.1056156)
Supplement: Supplementary Table 2 — Egger's test for FMA-UE. [file Table_2.DOCX]

Supplementary Material Table 2. Egger's test for FMA-UE

Std_Eff | Coef. Std. Err. t P>|t| [95% Conf. Interval]

slope | 6.246447 .8335381 7.49 0.000 4.561803 7.93109

bias | -.072526 .8627788 -0.08 **0.933** -1.816267 1.671215
