# Supplementary material for: EMG biofeedback combined with rehabilitation training may be the best physical therapy for improving upper limb motor function and relieving pain in patients with the post-stroke shoulder-hand syndrome: A Bayesian network meta-analysis
Source: Front Neurol. 2023 Jan 10;13:1056156. doi: 10.3389/fneur.2022.1056156 (PMC9873378; doi:10.3389/fneur.2022.1056156)
Supplement: Supplementary Table 3 — Egger's test for VAS. [file Table_3.DOCX]

Supplementary Material Table 3. Egger's test for VAS

Std_Eff | Coef. Std. Err. t P>|t| [95% Conf. Interval]

slope | -.7033147 .1820165 -3.86 0.000 -1.072828 -.3338015

bias | -2.234945 .8355602 -2.67 **0.011** -3.931223 -.5386681
